# A Novel Soft Peeling Serum Improves Overall Skin Appearance by Reducing Multiple Aging Signs in Chinese Women

**DOI:** 10.1111/jocd.71168

**Published:** 2026-09-17

**Authors:** Juanjuan Chen, Feixue Teng, Xingzuo Liu, Yue Wang, Lingling Sun, Yu Huang, Xiaomin Weng, Dan Wang, Shumei Li, Frederic Flament, Jing Li

**Affiliations:** ^1^ L'Oreal Research and Innovation Shanghai China; ^2^ L'Oreal Research and Innovation Paris France

**Keywords:** antiaging, gluconolactone, skin dullness, skin renewal, soft peeling

## Abstract

**Background:**

Skin aging involves complex structural and functional changes at the epidermal and dermal levels, often leading to dullness, fine lines, pigmentation, and wrinkles.

**Aims:**

This study evaluated a novel soft peeling serum designed to promote gentle exfoliation and target visible signs of aging in Chinese women.

**Patients/Methods:**

Two clinical trials were conducted. A dansyl chloride (DC) forearm test assessed the skin renewal effects of a soft peeling association (containing gluconolactone, lactic acid, salicylic acid, and surfactants) in 32 women aged 18–65. A 12‐week randomized controlled trial evaluated the antiaging efficacy of the full soft peeling serum, combining the soft peeling association with bifida ferment lysate and proxylane versus a benchmark serum in 124 women aged 18–45, stratified into younger and older groups.

**Results:**

In the DC test, the soft peeling association significantly reduced cumulative fluorescence scores and turnover days, indicating accelerated stratum corneum renewal. In the facial trial, the soft peeling serum significantly improved skin tone (translucency, dullness, hyperpigmentation), texture (smoothness, firmness, pores), and wrinkles in both age groups. Instrumental measurements confirmed increased hydration, improved elasticity, and reduced transepidermal water loss in both age groups. Compared with the benchmark, stronger improvements in skin tone and texture attributes were observed in the younger group with soft peeling serum application, while the older group showed stronger benefits in elasticity and crow's feet wrinkles reduction. No adverse events were reported, and all participants found the serum gentle on the skin.

**Conclusions:**

The soft peeling serum appears to be a gentle and effective formulation for improving multiple clinical signs of facial aging by enhancing the epidermal renewal. Compared to the benchmark serum, it provides rapid skin tone and texture improvements in younger subjects and progressively stronger improvements in elasticity and wrinkles in older subjects.

**Trial Registration:**

ClinicalTrials.gov: NCT07156071

## 1 Introduction

The skin serves as the body's primary interface with the external environment, providing essential protection against physical, chemical, and microbial insults [1]. Its outermost layer, the epidermis, is constantly renewing throughout life, maintaining the skin barrier through the proliferation and differentiation of basal keratinocytes [2]. This regenerative capacity is sustained by epidermal stem cells located in the basal layer, which give rise to new cells that migrate toward the surface and are eventually shed through desquamation [3]. However, with advancing age, the self‐renewal capacity of the epidermis declines, primarily due to a reduction in both the number and functional activity of these stem cells [4, 5]. This age‐related attenuation of epidermal turnover results in the retention of corneocytes, which in turn leads to skin dullness, rough texture, uneven tone, and compromised barrier integrity [6]. In addition to epidermal changes, aging exerts profound effects on the dermis, the supportive connective tissue layer beneath the epidermis. The dermis is rich in extracellular matrix (ECM) components, including collagen, elastin, and glycosaminoglycans, which confer structural integrity, elasticity, and hydration to the skin. With age, fibroblast function declines and ECM remodeling becomes dysregulated, leading to loss of dermal volume, reduced elasticity, and the deepening of wrinkles [7, 8, 9]. Therefore, effective antiaging strategies must target not only epidermal renewal but also dermal matrix preservation and regeneration. In response to these multifactorial changes, contemporary skincare regimens have increasingly focused on multitargeted approaches. These include formulations designed to stimulate epidermal turnover, restore skin barrier function, and promote ECM synthesis, aiming to holistically rejuvenate the skin and mitigate visible signs of aging [10, 11, 12, 13].

Topical hydroxy acids have long been utilized in dermatology and cosmetic science to accelerate epidermal renewal and improve signs of photoaging, such as glycolic acid and lactic acid. These acids have been reported to modulate desquamation enzymes (e.g., cathepsin D) and to affect the calcium‐mediated structure of corneodesmosomes, facilitating the breakdown of corneocyte adhesion and inducing exfoliation of the stratum corneum [14, 15, 16]. However, this exfoliating property could also bring adverse effects, such as skin discomfort, manifesting as erythema, stinging, peeling, or dryness, particularly in individuals with sensitive or compromised skin barrier function [17]. Polyhydroxy acids (PHAs) represent a next‐generation class of hydroxy acids characterized by multiple hydroxyl groups, which enhance their hydrophilic nature and confer additional humectant, antioxidant, and chelating properties [18]. Compared to lactic acids (AHAs), PHAs such as gluconolactone (GDL) penetrate more slowly due to their larger molecular size, resulting in minimal irritation and improved skin tolerance [18]. Complementing the exfoliative action of hydroxy acids, specific surfactants and emulsifiers can disrupt the lipid organization of the stratum corneum through non‐covalent interactions, facilitating the penetration of water‐soluble active compounds and promoting gentle desquamation.

In this study, a new soft peeling association was developed, containing gluconolactone (PHA), a lower concentration of AHA and salicylic acid (BHA), and also two mild surfactants, PEG‐7 glyceryl cocoate and polyglyceryl‐5 laurate. This association was designed to gently promote exfoliation and epidermal renewal without compromising skin barrier integrity. The inclusion of PHA, known for its high molecular weight and humectant properties, helps minimize irritation compared to traditional AHAs, making the formulation suitable for sensitive skin [17]. The DC labeling technique [19, 20, 21, 22], an established method for measuring stratum corneum renewal time, was used to validate the skin renewal efficacy of this soft peeling association. Based on this association, a gentle peeling serum was developed and further enhanced with proxylane—a well‐established antiaging ingredient demonstrated to stimulate glycosaminoglycan synthesis in the ECM and induce expression of collagen IV and collagen VII in the dermal‐epidermal junction (DEJ) [23, 24, 25]—alongside bifida ferment lysate, a probiotic‐derived active that reinforces the skin barrier and improves resilience [26]. This combination targets multiple pathways of skin aging, including stratum corneum turnover, dermal structure, and barrier function.

This study aimed to evaluate the efficacy of the novel soft peeling serum in promoting skin renewal and improving multiple signs of aging, with a particular emphasis on ensuring gentleness. Two distinct yet complementary clinical studies were performed. The first clinical study assessed the epidermal renewal effect of the soft peeling association. In the second clinical study, the soft peeling serum was evaluated for its comprehensive antiaging efficacy in both younger and older individuals, and its performance was compared against a benchmark serum containing an equivalent dose of bifida ferment lysate.

## 2 Materials and Methods

### 2.1 Clinical Study Design

This study comprised two components: a DC patch test (clinical study 1) and a randomized controlled clinical trial (clinical study 2). The DC test [19, 20, 21, 22], an established method for measuring stratum corneum renewal time, was designed to preliminarily assess the exfoliative efficacy of a soft peeling association composed of gluconolactone, lactic acid, salicylic acid, PEG‐7 glyceryl cocoate, and polyglyceryl‐5 laurate. The subsequent trial evaluated the safety, tolerability, and antiaging effects of a soft peeling serum formulated with the soft peeling association, bifida ferment lysate, and proxylane in both younger and older individuals. Both clinical studies followed a prospective, single‐center, double‐blind, randomized controlled design.

Clinical study 1 enrolled healthy Chinese women aged 18–65 years after providing written informed consent. The study protocol was approved by the Shanghai Ethics Committee for Clinical Research (SECCR2024‐228‐01). The trial was registered on ClinicalTrials.gov. A 5% (w/w) DC cream was prepared by dissolving thoroughly ground DC powder in molten white petrolatum. On the first day (Day 0), the technician will perform staining with DC on a defined 3 × 3 cm^2^ area of the inner forearm using an occlusive patch. On the second day (Day 1), approximately 24 h (at least 20 h) following patch application, the patch was removed, and baseline fluorescence of sites was graded under Wood's Light. After the clinical grading, investigational serums were applied to the corresponding sites.

From Day 2 to 21, the soft peeling association was applied once daily to the treated site, while the contralateral forearm served as an untreated control. Daily clinical grading of fluorescence intensity was performed by a dermatologist from Day 2 to 22 under Wood's Light. All procedures were conducted in a climate‐controlled environment (21°C ± 1°C, 50% ± 10% relative humidity). The outcomes were cumulative fluorescence score, calculated as the sum of daily scores, and epidermal turnover time, defined as the number of days required for fluorescence to decrease to a score of ≤ 0.5. Safety was evaluated by monitoring adverse events (AEs) throughout the study. Full methodological details, including inclusion and exclusion criteria, are provided in the Appendices S1 and S2. The fluorescence evaluation images are provided in the Figure S1.

Clinical study 2 was conducted between November 18, 2023, and March 1, 2024. The study enrolled Chinese women aged 18–45 years, stratified into two age‐based cohorts: younger group (18–29 years) and older group (30–45 years), with equal distribution between groups (1:1). Eligibility criteria for younger group included clinical signs such as reduced skin radiance, dullness, and mild nasolabial folds, graded between 2 and 6 on a modified Griffith scale (0 to 9 scale), and the presence of or acne‐related marks of post‐inflammatory hyperpigmentation (PIH) ≥ 2 mm in size. For the older group, inclusion required moderate to advanced signs of aging, including forehead wrinkles (grade > 2), crow's feet (grade > 2), and small folds on nasolabial zone (Grade 1–4), as evaluated by a trained dermatologist using the Skin Aging Atlas [27]. Additional inclusion criteria required participants to self‐report concerns such as dullness, rough texture, fine lines, or loss of radiance; to be regular users of facial serums and sunscreens; and to abstain from participating in any clinical or cosmetic trials in the preceding 3 months. Major exclusion criteria included dermatologic disorders in the test area (e.g., acne, rosacea, eczema), known hypersensitivity to cosmetic ingredients, recent excessive UV exposure, a history of skin cancer or melanoma, recent esthetic dermatologic procedures, or systemic medication use affecting skin biology within 6 months before enrollment. Full inclusion and exclusion criteria are provided in the Appendix S3. The study was conducted in accordance with the Declaration of Helsinki and the International Council for Harmonization Good Clinical Practice (ICH‐GCP) guidelines.

### 2.2 Procedure

Clinical study 2 comprised a 2‐week washout period followed by a 12‐week treatment phase. During the washout period, participants were instructed to discontinue all personal skincare products and instead use a standardized set of auxiliary products, including a cleanser, moisturizer, and broad‐spectrum sunscreen, provided by the study team. On the first visit, participants washed their face with the standard cleanser and underwent a 30‐min acclimatization in a controlled environment (21°C ± 1°C temperature; 45% ± 5% relative humidity). Baseline clinical assessments were then conducted by a dermatologist and a trained technician. Participants were randomly assigned in a 1:1 ratio to receive either the soft peeling serum (containing the soft peeling association, proxylane and bifida ferment lysate) or the benchmark serum (containing an equivalent dose of bifida ferment lysate). The test and control products were identical in appearance, smell, color, and texture, ensuring blinding throughout the study. Randomization was carried out using a computer‐generated random number table. Both participants and investigators were blinded to group allocation.

Throughout the 12‐week treatment period, the subjects were instructed to apply the investigational serum twice daily (morning and evening), following the standard regimen of cleanser and moisturizer; sunscreen was applied in the morning only. Compliance and skin tolerance were monitored daily, with participants instructed to record any adverse sensations or signs of intolerance. Clinical evaluations were conducted at predefined time points: baseline (T0), and Weeks 4 (T4w), 8 (T8w), and 12 (T12w). All follow‐up assessments were performed in the same controlled environment by the same clinical personnel to minimize inter‐rater and environmental variability.

### 2.3 Endpoints and Assessment

The primary efficacy endpoints included clinical parameters related to skin quality, skin tone, and wrinkles, evaluated by the same board‐certified dermatologist throughout the study. The core study conclusions are based on quantitative clinical and instrumental data, with photographs serving only as supportive qualitative documentation. Safety and gentleness evaluation included dermatologist‐conducted assessments of physical signs of irritation or discomfort, alongside participant‐reported symptoms and tolerability feedback collected throughout the study period.

Assessments were guided by the Skin Aging Atlas and a modified Griffiths scale (0 to 9 scale) [28], as detailed in the Appendices S4–S6. Antiaging parameters included the severity of crow's feet wrinkles, forehead wrinkles, global wrinkles, small folds on the nasolabial zone, and overall fine lines. Skin quality attributes encompassed visual and tactile evaluations of smoothness, softness, elasticity, firmness, plumpness, and cheek pore visibility. Skin tone measures included radiance, translucency, overall hyperpigmentation, fairness, evenness, and dullness. For participants in the younger group, PIH of acne marks and lesion size were additionally assessed. Standardized facial imaging capturing frontal and bilateral views was performed at each visit using the VISIA‐CR system (Canfield Scientific, USA). Objective skin measurements included hydration (Corneometer, Courage & Khazaka, Germany), barrier function assessed by transepidermal water loss (TEWL; Vapometer, Delfin Technologies, Finland), and skin elasticity (Cutometer Dual MPA 580, Courage & Khazaka, Germany) (Appendix S7). Participants also completed a structured questionnaire at each timepoint to assess subjective perceptions of efficacy and tolerability (Appendix S8).

### 2.4 Statistical Analysis

In clinical study 1, statistical comparisons of cumulative fluorescence scores and epidermal turnover days were performed using a paired *t*‐test for normally distributed data, or the Wilcoxon signed‐rank test for non‐normally distributed data. In clinical study 2, normality was assessed using the Shapiro–Wilk test. Changes from baseline in efficacy endpoints were analyzed using one‐way ANOVA for normally distributed variables or the Wilcoxon signed‐rank test otherwise. Between‐group differences at each timepoint were evaluated using the independent samples *t*‐test or the Mann–Whitney U test, depending on the data distribution. For categorical outcomes derived from participant questionnaires, the frequencies and proportions of responses indicating “completely agree” and “somewhat agree” were calculated. All statistical tests were two‐sided, and *p*‐values of < 0.05 were considered statistically significant. Analyses were conducted using SPSS software, version SPSS25.0 (IBM Corp., Armonk, NY, USA).

## 3 Results

### 3.1 Skin Renewal Effects of the Soft Peeling Association

In clinical study 1, 34 participants were enrolled, of whom 32 completed the study. The average age of participants was 52.63 ± 10.06 years. The mean cumulative fluorescence score at the treated site was 58.42 ± 2.25, significantly lower than 71.59 ± 2.09 at the control site (*p* < 0.001) (Figure S2). Similarly, the mean epidermal turnover time was 19.38 ± 0.59 days in the treated area versus 21.69 ± 0.45 days in the control (*p* = 0.001).

### 3.2 Anti‐Aging Efficacy of the Soft Peeling Serum

In clinical study 2, a total of 133 participants were enrolled, of whom 124 completed the 14‐week trial and were included in the efficacy analysis set. Nine participants withdrew due to personal reasons unrelated to the intervention. Of those who completed the study, 63 participants were allocated to the soft peeling serum group and 61 to the benchmark serum group. The distribution of younger and older subgroups was comparable between arms, with 31 younger and 32 older participants in the soft peeling serum group, and 30 and 31 participants, respectively, in the benchmark group. All participants were Asian females. The mean age of younger participants was 26.74 ± 2.52 years in the soft peeling serum group and 27.50 ± 2.35 years in the benchmark group. Among older participants, the mean age was 41.66 ± 2.82 years and 41.77 ± 2.49 years in the respective groups (Table 1).

**TABLE 1:** Baseline characteristics of participants in clinical study 2.

Variables	Younger (*N* = 61)		Older (*N* = 63)	
	Soft peeling serum (*N* = 31)	Benchmark serum (*N* = 30)	Soft peeling serum (*N* = 32)	Benchmark serum (*N* = 31)
Female, *n* (%)	31 (100.0%)	30 (100.0%)	32 (100.0%)	31 (100.0%)
Age, years, mean ± SD	26.74 ± 2.52	27.50 ± 2.35	41.66 ± 2.82	41.77 ± 2.49
Skin type, *n* (%)				
Dry	4 (12.9%)	3 (10.0%)	13 (40.6%)	13 (41.9%)
Normal	2 (6.5%)	2 (6.7%)	2 (6.3%)	4 (12.9%)
Oily	5 (16.1%)	6 (20.0%)	3 (9.4%)	1 (3.2%)
Mix	20 (64.5%)	19 (63.3%)	14 (43.8%)	13 (41.9%)

Abbreviation: SD, standard deviation.

### 3.3 Efficacy Texture Improvement Efficacy

**FIGURE 1:**
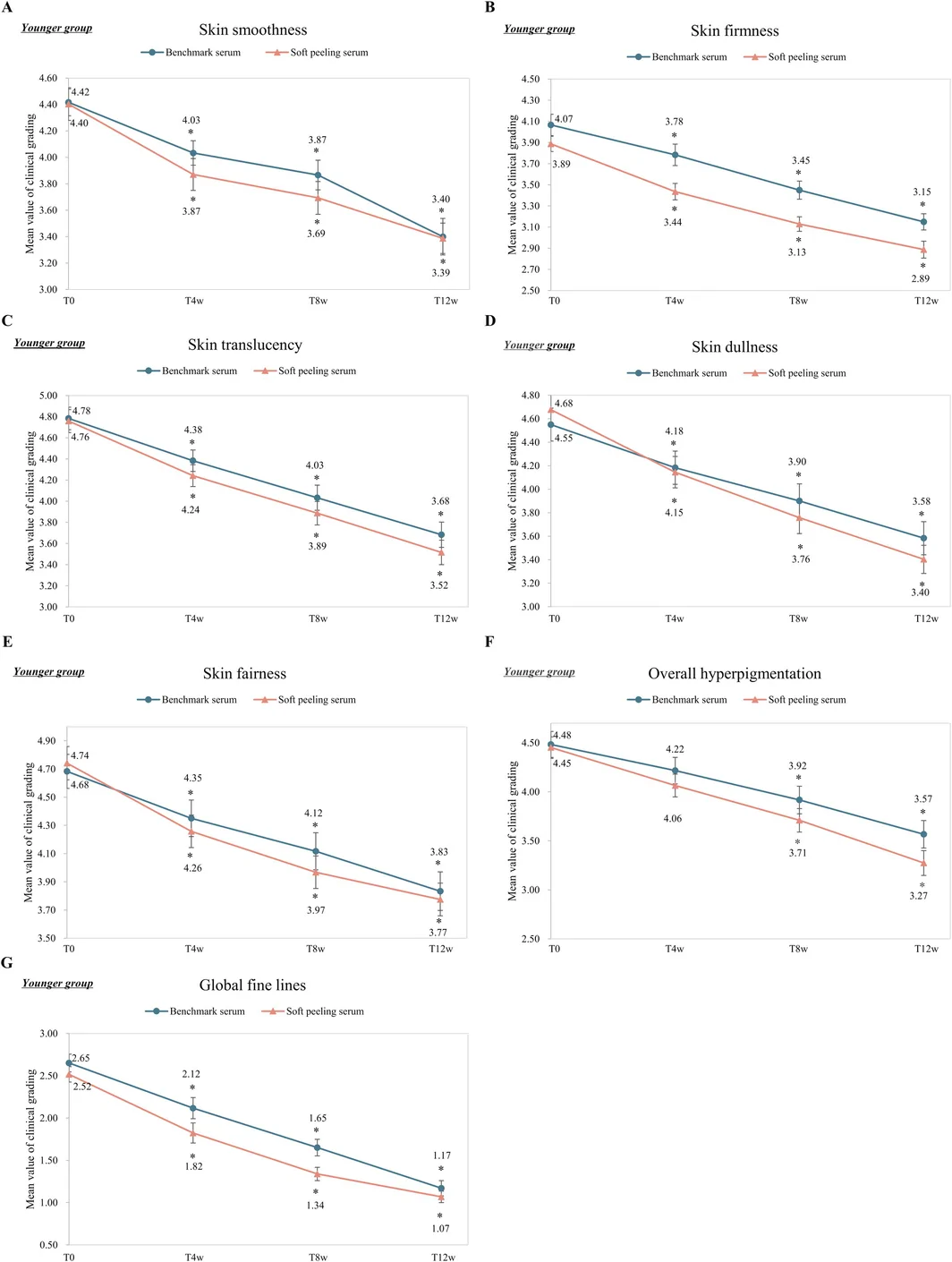
Trends in skin attributes over time in the younger population. * indicates a statistically significant difference with *p* < 0.05.

In the younger group, a broad and rapid improvement in skin texture parameters was observed. Significant reductions from baseline were observed by Week 4 in scores for skin plumpness, elasticity, softness, smoothness, and firmness (all *p* < 0.05). By Week 12, mean reductions reached 22.88%, 16.91%, 24.29%, 23.08%, and 25.73%, respectively (all *p* < 0.05). Cheek pore scores also showed significant improvement by Week 8 (*p* < 0.05), with a 19.90% reduction maintained through Week 12 (*p* < 0.05). Compared with the benchmark serum, the soft peeling serum yielded significantly greater improvements in skin smoothness (*p* = 0.042) and firmness (*p* = 0.006) at Week 4 (Figure 1 and Figure S3).

**FIGURE 2:**
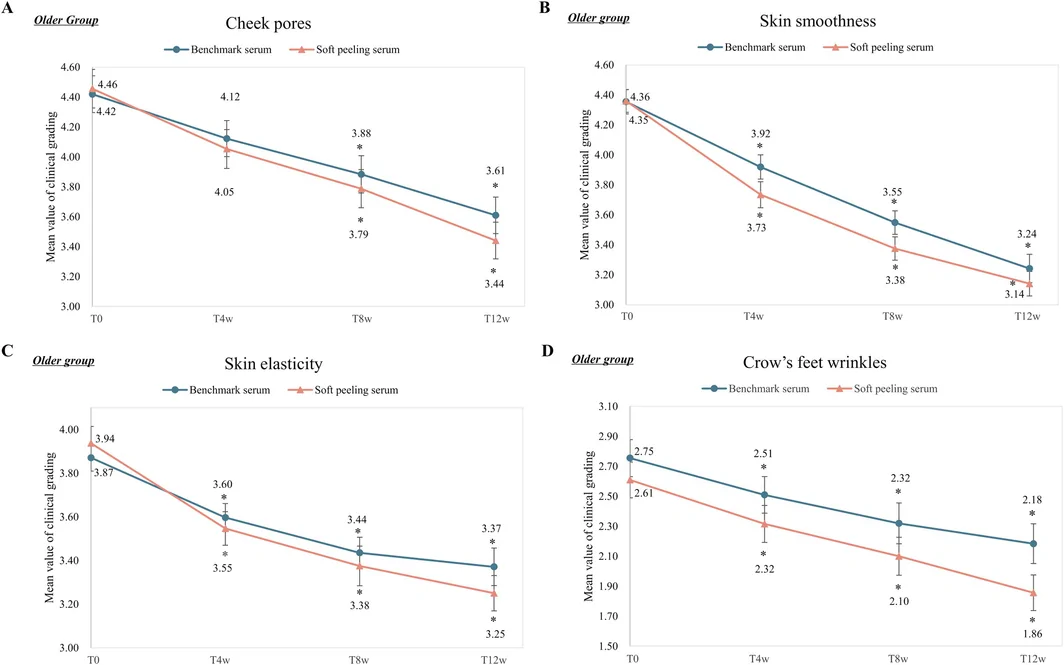
Trends in skin attributes over time in the older population. * indicates a statistically significant difference with *p* < 0.05.

Among older participants, similar improvements were observed. Significant reductions from baseline were noted by Week 4 in firmness, plumpness, elasticity, softness, and smoothness (all *p* < 0.05), with corresponding improvements of 21.52%, 21.74%, 17.46%, 24.90%, and 27.96% by Week 12 (all *p* < 0.05). Cheek pore scores improved significantly at Week 8 (*p* = 0.001), with a 22.79% reduction sustained at Week 12 (*p* < 0.001). Compared with the benchmark serum, the soft peeling serum demonstrated significantly superior improvement in cheek pore scores at both Week 8 (*p* = 0.049) and Week 12 (*p* = 0.013), as well as in skin smoothness at Week 4 (*p* = 0.020) and skin elasticity at Week 12 (*p* = 0.015) (Figure 2 and Figure S4).

### 3.4 Skin Tone Improvement Efficacy

**FIGURE 3:**
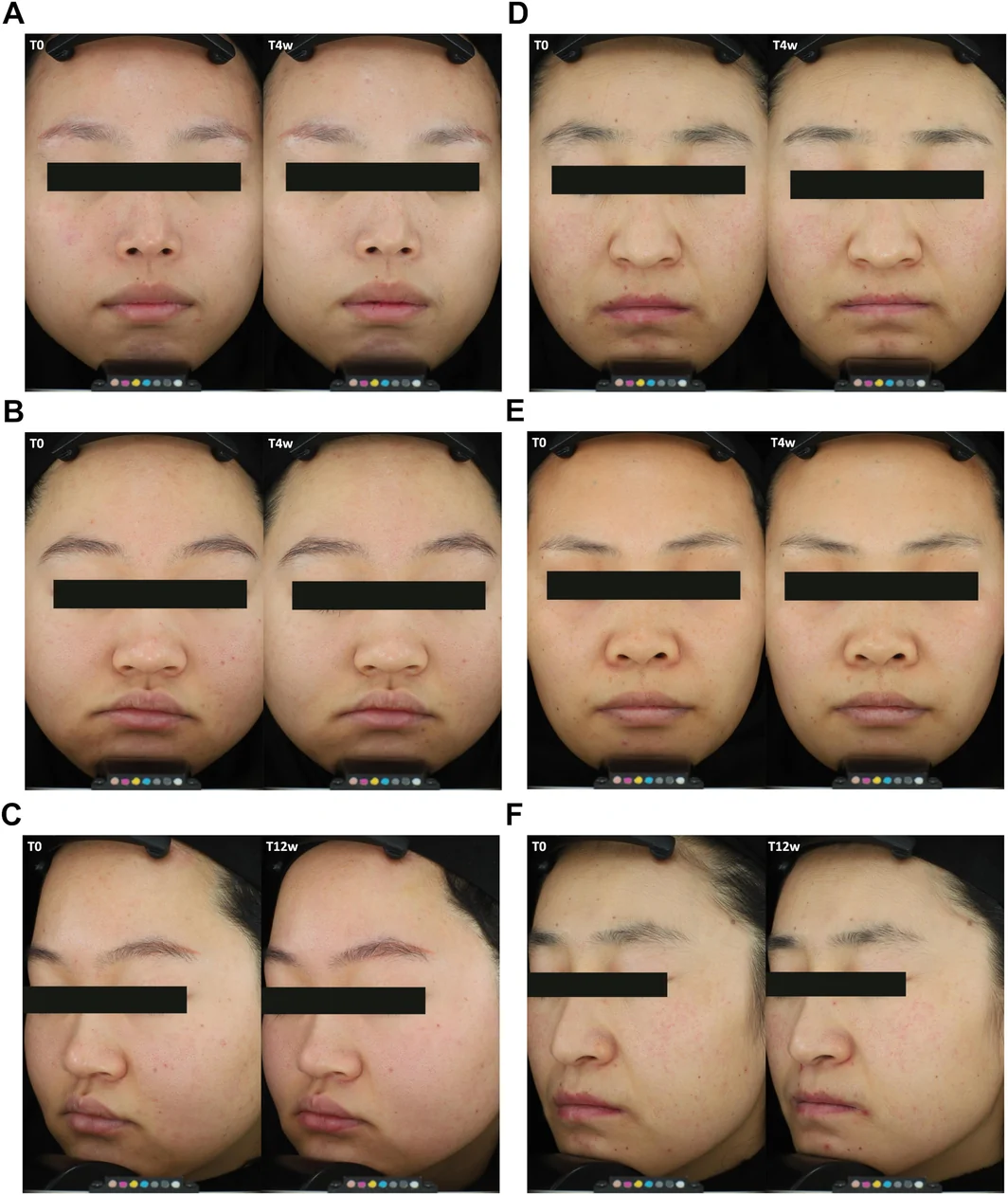
Representative case images from the younger population. (A) Skin translucency in the soft peeling serum group; (B) Skin dullness in the soft peeling serum group; (C) Overall hyperpigmentation in the soft peeling serum group; (D) Skin translucency in the benchmark serum group; (E) Skin dullness in the benchmark serum group; (F) Overall hyperpigmentation in the benchmark serum group.

In the younger group, improvements from baseline were evident as early as Week 4 across several attributes, including skin radiance, tone evenness, translucency, fairness, dullness, PIH of acne mark (all *p* < 0.05). By Week 12, respective improvements reached 19.93%, 19.27%, 26.10%, 20.41%, 27.24%, 43.01%. Overall hyperpigmentation showed significant reductions at Weeks 8 and 12 (both *p* < 0.05), with a 26.45% improvement observed by Week 12. Compared with the benchmark serum, the soft peeling serum demonstrated significantly greater efficacy in improving skin translucency, dullness, and fairness at Week 4 (all *p* < 0.05), with continued superiority in dullness at Week 8 and in both dullness and hyperpigmentation at Week 12 (all *p* < 0.05) (Figures 1 and Figure 3, and Figure S3).

**FIGURE 4:**
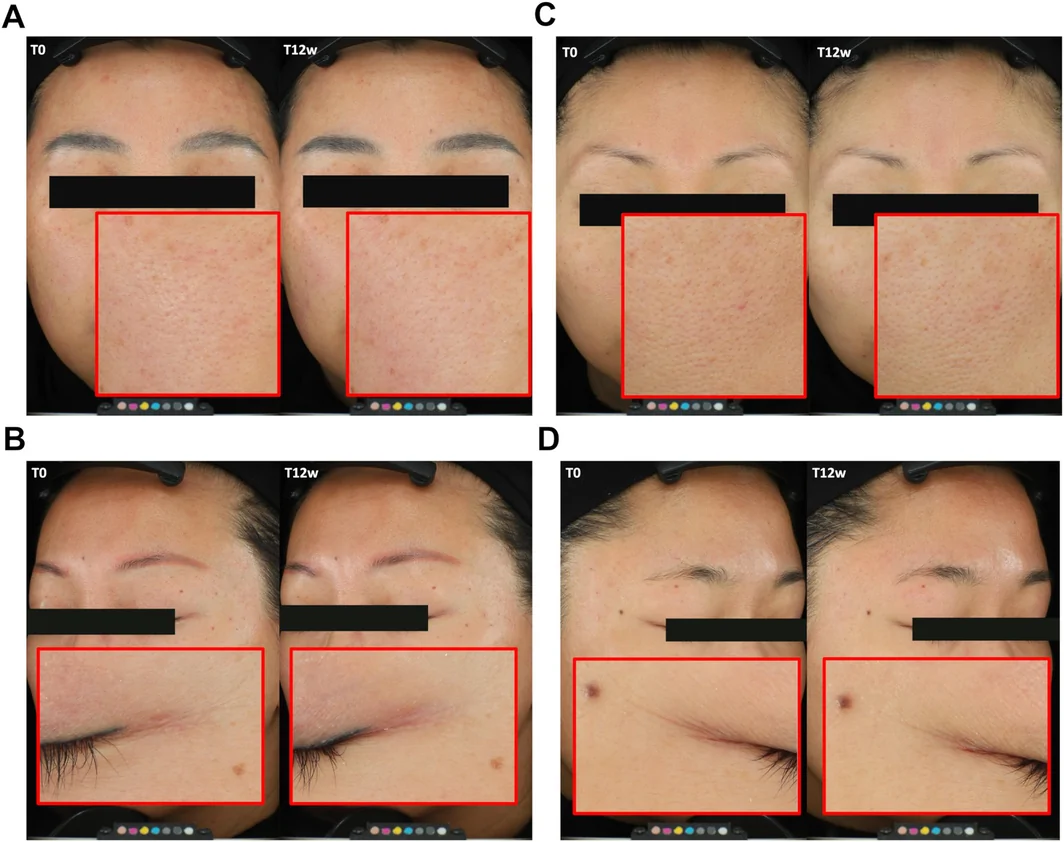
Representative case images from the older group. (A) Cheek pores in the soft peeling serum group; (B) Crow's feet wrinkles in the soft peeling serum group; (C) Cheek pores in the benchmark serum group; (D) Crow's feet wrinkles in the benchmark serum group.

In the older group, significant enhancements in skin radiance, translucency, fairness, and dullness were observed by Week 4 (all *p* < 0.05), with improvements of 19.87%, 22.12%, 18.46%, and 22.15%, respectively, by Week 12. Skin tone evenness and overall hyperpigmentation exhibited significant improvements beginning at Week 8 (both *p* < 0.05), with Week 12 improvement rates reaching 15.96% and 17.81%, respectively (Figures 2 and 4, Figure S4).

### 3.5 Wrinkle Improvement Efficacy

In the younger group, significant wrinkle improvements were also observed by Week 4 in forehead wrinkles, small folds on the nasolabial zone, and global fine lines (all *p* < 0.05), with reduction rates of 44.72%, 50.68%, and 57.56%, respectively, by Week 12. Crow's feet wrinkles and global wrinkles showed significant reductions beginning at Week 8 (both *p* < 0.05), with 12‐week reductions of 41.99% and 19.12%, respectively (both *p* < 0.05). Compared with the benchmark serum, the soft peeling serum demonstrated significantly greater efficacy in improving global fine lines at Week 4 (*p* < 0.05) (Figure 1 and Figure S3).

Among older participants, significant improvements in forehead wrinkles, nasolabial folds, and global fine lines were also observed by Week 4 (all *p* < 0.05), with reductions of 39.42%, 35.97%, and 48.26%, respectively, by Week 12. Crow's feet wrinkles showed significant improvement at Week 8 (*p* = 0.015), with a 28.86% reduction achieved by Week 12 (*p* < 0.001). When compared with the benchmark serum, the soft peeling serum produced a statistically greater reduction in crow's feet wrinkles at Week 12 (*p* = 0.036) (Figure 2 and Figure S4).

### 3.6 Skin Hydration, Barrier Function, and Elasticity

Improvements in skin barrier function, as measured by TEWL via Vapometer, and in skin elasticity, as assessed by Cutometer R2, were observed from Week 4 onward (both *p* < 0.05). Skin hydration, measured by Corneometer, showed significant enhancement by Week 12 (both *p* < 0.05). In younger participants, the respective improvement rates at Week 12 were 31.16% for TEWL, 19.71% for elasticity, and 12.89% for hydration. Corresponding improvements in the older group were 27.80%, 17.67%, and 12.06%, respectively (Figure S5).

### 3.7 Gentleness and Safety

Self‐assessment questionnaires indicated that 100% of participants in both younger and older groups rated the soft peeling serum and the benchmark serum as gentle on the skin after 4 weeks of use. No AEs or serious AEs were reported in either treatment group throughout the DC test and 14‐week study period.

## 4 Discussion

This study employed distinct but complementary study designs to evaluate key attributes of a novel soft peeling formulation. While many previous studies have examined either the epidermal renewal efficacy by different ingredients working on skin exfoliation or the improvement of facial aging signs by different formulas containing a variety of antiaging ingredients in isolation, the present study sought to sequentially assess skin renewal and observed clinical facial aging parameters, exploring potential holistic skin benefits in both younger and older Chinese women.

The DC test was selected to specifically quantify stratum corneum renewal kinetics of a novel soft peeling association combining gluconolactone (PHA), lower concentration of lactic acid (AHA), salicylic acid (BHA), and two mild surfactants (PEG‐7 glyceryl cocoate and polyglyceryl‐5 laurate). Results demonstrated that the newly developed peeling association significantly accelerated epidermal renewal, as shown by a marked reduction in both cumulative fluorescence score and turnover days in the DC test. These findings suggest the association's potential to support controlled exfoliation of the stratum corneum. The observed desquamation effect can be attributed to the plausible combined action of gluconolactone, lactic acid, and salicylic acid, agents known to disrupt corneocyte cohesion [17], alongside mild surfactants, which support barrier‐friendly exfoliation. Importantly, no AEs were reported, confirming a favorable safety profile for this soft peeling association.

Building on these initial renewal observations, a multi‐active serum incorporating this peeling association plus proxylane (widely recognized to stimulate GAG synthesis and support dermal matrix integrity [29]) and bifida ferment lysate (reported to reinforce barrier function and repair [26]) was further evaluated for its clinical performance in Chinese females aged 18–45 years over 12 weeks. This clinical design aimed to observe broader facial aging outcomes under typical use conditions, enabling evaluation of the serum's performance against multiple signs of facial aging. In clinical study 2, the age range was intentionally defined to minimize potential confounding from significant hormonal fluctuations characteristic of perimenopause and menopause, thereby establishing a relatively homogeneous baseline.

Over the 12‐week treatment period, the soft peeling serum significantly improved skin tone, texture, and wrinkle attributes in both younger and older groups. Observed visual improvements in skin radiance, transparency, and pigmentation may be mechanistically linked to the enhanced desquamation of melanin‐containing corneocytes, whereas improvements in skin firmness, elasticity, and wrinkles could potentially reflect both epidermal turnover and long‐term dermal support. These observations suggest that such a multi‐targeted formulation may offer complementary benefits across broad age groups (aged 18–45).

Compared with the benchmark serum, the soft peeling serum demonstrated superior efficacy across several parameters. In the younger group, participants exhibited earlier and more pronounced improvements in skin translucency, fairness, dullness, smoothness, and global fine lines. These observed trajectory changes align with the concept that accelerating epidermal renewal helps address early surface dullness, which is frequently reported as a primary esthetic concern among younger Asian populations [30, 31]. Compared with the benchmark serum, the benefits of the soft peeling serum in the older group appeared more gradually, which would be conceptually consistent with the slower biological turnover and deeper structural changes associated with aging skin. For instance, a numerical reduction in cheek pore size that reached statistical significance was noted in the soft peeling serum group compared to the benchmark as early as Week 4, with sustained trends through Week 12. Such effects might be facilitated by ongoing surface smoothing, potentially supported by the incorporated BHA [32, 33]. More gradual but substantial improvements over the benchmark were observed in skin elasticity and crow's feet wrinkles, reaching statistical significance at Week 12. This extended onset timeline is primarily driven by the long‐term synergistic action of proxylane and the soft peeling association within the formulation. Specifically, while the soft peeling complex facilitates continuous epidermal renewal, proxylane promotes ECM reorganization and restores skin firmness by stimulating glycosaminoglycan synthesis, thereby mediating progressive structural anti‐wrinkle efficacy.

The soft peeling serum demonstrated an ability to enhance epidermal turnover and improve key skin biophysical properties, as evidenced by sustained improvements in skin hydration, TEWL, and elasticity across 18–45 aged Chinese females over the 12‐week treatment period, indicating progressive support of skin function and mechanical resilience. Mechanistically, the exfoliative action of the soft peeling association, driven by gluconolactone, lactic acid, and salicylic acid, facilitates controlled desquamation of the stratum corneum and cell renewal [34].

The formulation demonstrated an excellent safety profile, with no reports of AEs or clinically significant skin irritation in either trial, supported by high (100%) self‐reported gentleness ratings in questionnaires. These findings are particularly noteworthy given the inclusion of exfoliating acids, which are often associated with stinging, dryness, or erythema in traditional chemical peeling formulations [17]. In contrast, the current formulation, incorporating PHAs and mild surfactants, appears to balance efficacy with tolerability, making it suitable for daily use. This favorable safety profile supports its clinical relevance as a gentle yet effective daily cosmetic intervention.

This study has several limitations. First, both the relatively small sample size and reliance on simple *t*‐tests or Mann–Whitney U tests may limit the statistical power. Second, the lack of an active‐comparator control in clinical study 2 limits direct comparisons of clinical superiority. Finally, the study population comprised exclusively Chinese women; replication in other ethnicities or phototypes would strengthen generalizability.

## 5 Conclusion

This study systematically evaluated the safety, tolerability, and antiaging efficacy of a soft peeling serum formulated with a polyhydroxy acid‐based peeling association, proxylane, and bifida ferment lysate. By combining mechanistic assessment of epidermal renewal with 12‐week clinical evaluations, the study observed favorable performance across both younger and older Chinese women, accompanied by an excellent safety profile, high tolerability, and a consensus among participants that it was gentle on the skin. Nonetheless, given the limited sample size and single‐ethnicity study population, further large‐scale studies are warranted to confirm these results and generalize their applicability.

## Author Contributions

Jing Li, Juanjuan Chen and Xingzuo Liu, Dan Wang and Shumei Li contributed to the conceptualization of this research. Feixue Teng, Yue Wang, Lingling Sun, Yu Huang and Xiaomin Weng contributed to the soft peeling association concept. Feixue Teng designed the soft peeling serum. Juanjuan Chen managed the clinical studies and wrote the paper. Jing Li and Frederic Flament reviewed the paper.

## Ethics Statement

Ethical approval was granted by the Shanghai China‐norm Quality Technical Service Co. Ltd. Ethics Committee (FSEC 2023–104‐01), and all participants provided written informed consent prior to enrolment.

## Consent

Consent for publication of identifiable images or photographs has been obtained from the individuals concerned.

## Conflicts of Interest

All the authors are employees of L'Oréal.

## Supporting information


**Appendix S1:** Inclusion and exclusion criteria in clinical study 1.
**Appendix S2:** Dansyl chloride test protocol.
**Appendix S3:** Inclusion and exclusion criteria in clinical study 2.
**Appendix S4:** Size of an isolated acne mark.
**Appendix S5:** Attributes in Griffith scale (0 to 9 scale).
**Appendix S6:** Attributes of the physical symptom in scale (0 to 4 scale).
**Appendix S7:** Diagram 1 skin measurement.
**Appendix S8:** Self‐reported questionnaire.
**Figure S1:** The fluorescence evaluation images in dansyl chloride patch test.
**Figure S2:** Cumulative fluorescence scores in clinical study 1.
**Figure S3:** Trends in skin attributes over time in the younger group.
**Figure S4:** Trends in skin attributes over time in the older group.
**Figure S5:** Trend in skin hydration, TEWL, and elastic properties trend.
**Table S1:** Size of acne mark in clinical study 2.
**Table S2:** Skin tone attributes in clinical study 2.
**Table S3:** Skin quality attributes in clinical study 2.
**Table S4:** Wrinkle attributes in clinical study 2.

## Data Availability

The raw data that support the findings of this study are available from the corresponding author upon reasonable request.
