# Supplementary material for: A Novel Soft Peeling Serum Improves Overall Skin Appearance by Reducing Multiple Aging Signs in Chinese Women
Source: J Cosmet Dermatol. 2026 Sep 17;25(9):e71168. doi: 10.1111/jocd.71168 (PMC13586542; doi:10.1111/jocd.71168)
Supplement: Supplementary file 1 [file JOCD-25-e71168-s001.docx]

**Supplementary Appendix**

**A Novel Soft Peeling Serum Improves Overall Skin Appearance by Reducing Multiple Skin Aging Signs in Chinese Women**

**Contents**

**Supplementary Appendix A. Inclusion and exclusion criteria in clinical study 1**

**Supplementary Appendix B. Dansyl chloride test protocol**

**Supplementary Appendix C. Inclusion and Exclusion Criteria in clinical study 2**

**Supplementary Appendix D. Size of an isolated acne mark**

**Supplementary Appendix E.** **Attributes in Griffith scale (0 to 9 scale)**

**Supplementary Appendix F. Attributes of the physical symptom in scale (0 to 4 scale)**

**Supplementary Appendix G. Diagram 1 skin measurement**

**Supplementary Appendix H. Self-reported questionnaire**

**Table S1.** **Size of acne mark in clinical study 2**

**Table S2.** **Skin tone attributes in clinical study 2**

**Table S3.** **Skin quality attributes in clinical study 2**

**Table S4. Wrinkle attributes in clinical study 2**

**Figure S1. The fluorescence evaluation images in dansyl chloride patch test.**

**Figure S2. Cumulative fluorescence scores in clinical study 1**

**Figure S3. Trends in skin attributes over time in the younger group**

**Figure S4. Trends in skin attributes over time in the older group**

**Figure S5. Skin hydration, TEWL and elastic properties trend**

**Supplementary Appendix A. Inclusion and exclusion criteria in the clinical study 1**

1. Inclusion criteria
1) Chinese physically healthy female participants, aged between 18 and 65 years old;
2) All skin types (including dry, normal, oily, and combination);
3) Have no significant skin defects, scars, tattoos, protrusions, open wounds, or other factors that would affect the evaluation on the inner side of the forearm skin;
4) Have no severe allergic reactions to the testing reagents containing dansyl chloride;
5) Agree to avoid swimming, prolonged sun exposure, sauna, and steam baths during the study period;
6) Agree to discontinue the use of any other cleansing and skincare products on the forearm during the study period;
7) Willingness to read, write and sign an informed consent form to demonstrate willingness and ability to participate;
8) Willingness to comply with all requirements of the research protocol;
9) Have no reason assessed by the investigators as unsuitable for participation in the testing;
10) Intends to complete the study and is willing and able to follow all study instructions.
11) Subjects who could comply with standards during the whole study that refrain from the use of towel, bath ball or any other bath sponges on the forearm.
2. Exclusion criteria
1) Undergoing dermatological treatment or those who have taken whitening or anti-aging dietary supplements within the past month;
2) Have allergies to skincare products, soap, alcohol, fragrances, or medications;
3) Have used antihistamines within the past week or immunosuppressants within the past month;
4) Have applied any anti-inflammatory drugs to the test area within the past two months;
5) Have skin conditions such as psoriasis, eczema, dermatitis, skin cancer, etc.;
6) Insulin-dependent diabetes patients;
7) Undergoing treatment for asthma or other chronic respiratory diseases;
8) Have received cancer chemotherapy within the past 6 months;
9) With immunodeficiency or autoimmune diseases;
10) Breastfeeding or pregnant women;
11) Have undergone bilateral mastectomy and bilateral axillary lymph node dissection;
12) With facial erythema, follicular papules, sunspots, wounds, abrasions, tattoos, acne, or any other conditions that may affect the evaluation of test results;
13) With high sensitivity;
14) Have participated in intense outdoor activities or travel that may cause skin damage due to sun exposure within the month prior to the test;
15) Concurrently participating in another clinical trial or who have participated in a facial clinical trial within the past three months;
16) Have applied vitamin A analogues, alpha-hydroxy acids, salicylic acid, hydroquinone, or prescription drugs (antibiotics, vitamin A analogues, alpha-hydroxy acids, and corticosteroids) within the past six months, or oral contraceptives (continuation is allowed if the same contraceptive has been used consistently for the past six months);
17) PI or experts who believe that there are other medical reasons that may affect the test results.

**Supplementary Appendix B. Dansyl Chloride Test Protocol**

Before initiation of any study procedures, the study protocol was explained in detail to each subject by the principal investigator or a designated study staff member. Written informed consent was obtained in accordance with the procedures outlined in the “Informed Consent” section. Only participants meeting all inclusion criteria and none of the exclusion criteria were enrolled. All site assessments were performed under controlled environmental conditions (21±1°C and 50±10% relative humidity), including a standardized acclimatization period prior to each visit. The study was conducted over a maximum of 23 days, using designated test areas on the inner forearms (each measuring 3 cm × 3 cm). On day 0 (D0), the technician applied dansyl chloride to the designated skin areas using a patch applicator. The reagent was secured with an occlusive, waterproof adhesive and left in place for approximately 24 hours. On day 1 (D1), after at least 20 hours of staining, the patches were removed and standardized photographs were taken. The application sites were then evaluated under Wood’s light, and fluorescence was graded by a trained dermatologist. Following this baseline assessment, the test product (soft peeling association) was applied to the designated area. From day 2 to day 21 (D2-D21), daily clinical grading and image capture were conducted by the study team prior to each application of the test product. After each application, participants were asked to report any adverse reactions and to provide detailed descriptions of symptoms, including onset, severity, and duration. On the final day (D22), a concluding clinical assessment and image capture were performed to evaluate the endpoint parameters.

**Supplementary Appendix C. Inclusion and exclusion criteria in the clinical study 2**

1. Inclusion criteria
1) Chinese female with 18-45 years old (evenly split among Younger group (18-29 years old) and Older group 30-45 years old);
2) All skin types (dry, normal, oily or mixed);
3) Currently with lacking of radiance, rough, dullness and with fine lines or wrinkles on facial skin by self-claimed;
4) Younger group (18-29 years old): Presenting with corresponding severity for the attribute evaluated by Dermatologist included:
i. Skin smoothness, radiance, dullness: 2≤grade≤6 (0-9 scale);
ii. Size of acne mark (PIH): 2mm≤size;
iii. Small folds on nasolabial zone: 1≤grade≤3 (Skin Aging Atlas, of 0 to 6 on Page 56 to 57);

Mature aging (30-45 years old): Presenting with corresponding severity for the attribute evaluated by Dermatologist included:

i. Skin smoothness, radiance, dullness: 4≤grade≤6 (0-9 scale);
ii. Small folds on nasolabial zone: 1≤grade≤4 (Skin Aging Atlas, of 0 to 6 on Page 56 to 57);
iii. Forehead wrinkles: 2≤grade (Skin Aging Atlas, of 0 to 8 on Page 32 to 33);
iv. Crow’s feet wrinkles: 2≤grade (Skin Aging Atlas, of 0 to 6 on Page 40 to 41);
5) Regular user of serum and sunscreen products;
6) Did not participate any clinical test or cosmetic product test on skin within 3 months;
7) No disagreement of dermatologist because of other reasons that exclude the participation of the volunteer.
8) In general, good health at the time of the study;
9) Willing and able to participate as evidenced by signing of informed consent & photo release form;
10) Must be willing to comply with all study protocol requirements (pay attention to: only use the skin care products provided during the study, not take topical or oral treatment like retinol, hormone, anti-oxidant health-care products which may affect the test product).
2. Exclusion criteria
   1) Pregnant or breast-feeding woman or woman planning pregnancy during the study.
   2) Subject deprived of rights by a court or administrative order.
   3) Major subject to a guardianship order.
   4) Subject residing in a health or social care establishment.
   5) Patient in an emergency setting.
   6) Volunteer with a skin disease in the test areas as well as skin allergy (particularly e.g., acne, rosacea, eczema).
   7) Volunteer presenting a stable or progressive serious disease (per investigator's assessment).
   8) Immuno-compromised subject.
   9) Subject with history of allergy to cosmetic or personal care products or ingredients.
   10) Subject presenting excessive exposure to sunlight or UV radiation (investigator's assessment).
   11) Subject with history of sunlight sensitivity or allergies.
   12) Subjects regularly practicing aquatic or nautical sports.
   13) Subjects regularly attending a sauna.
   14) Subject with physical highly sensitive constitution.
   15) Subject with cardiovascular or circulatory history.
   16) Subject with a history of skin cancer or malignant melanoma.
   17) Subject with history of medical beauty treatment and taking part in anti-aging studies in the last 3 months before study.
   18) Intake of antihistamines, antibiotics, corticosteroids, non-steroidal anti-inflammatories or immune-suppressants in the last 6 months before study.

**Supplementary Appendix D. Size of an isolated acne mark**

**Supplementary Appendix E. Attributes in Griffith scale (0 to 9 scale)**

| **No.** | **Atlas** | **Method** | **Best Condition** | **Slight** | **Moderate** | **Worst Condition** |
| --- | --- | --- | --- | --- | --- | --- |
|  |  |  | **Score 0** | **Score 1~3** | **Score 4~6** | **Score 7~9** |
| 1. | Skin Radiance | Visual | Extremely radiant, luminous or glowing appearance all over the tested area | Adequate to acceptable radiance, most area with visibly radiant and glowing appearance | Perceivable to slight radiance | Very slight radiance or extreme dull looking, skin is heavily dull and matte, no luster on face |
| 2. | Skin tone Evenness | Visual | Very even tone: no detractions | Slight uneven skin tone: minor detractions covering less than 15% | Somewhat uneven skin tone: detractions covering less than 50% | Uneven, discolored appearance (brown and/or red colors), detractions covering more than 50% |
| 3. | Skin Translucency | Visual | Very translucent, clarity skin and bright skin, without any blemishes | Adequate to acceptable translucent | Perceivable to slight translucent | Lacking of translucency, opacity, dullness, yellowish |
| 4. | Skin Dullness | Visual | Skin is radiant and bright, the dullness is barely visible | Mild dullness: Minimal dullness or lack of radiance. Skin may appear slightly lackluster or less vibrant than usual | Moderate dullness: Noticeable dullness or lack of brightness. Skin may have an uneven tone, texture, or show signs of fatigue | Severe dullness: Pronounced dullness or lack of radiance. Skin may look tired, sallow, or lackluster. Uneven skin tone and texture may be more apparent |
| 5. | Skin Firmness | Visual | Looks very  tight and firm. | Fairly tight, with the assessed area firm. | Perceptible firm. | Very little firm or very slack in the assessed area. |
| 6. | Skin plumpness | Visual | Very plump, no fine line reflection at all, hydrated skin | Adequate to acceptable plump | Perceivable to slight plump | Very wizened, severe fine line reflection, lack of moisture |
| 7. | Skin Elasticity | Tactile | Skin feels toned, dense and resilient, skin can be snapped back immediately, there are no pressing traces. | Elastic but slight flabby. | Moderate flabby. | Skin feels pliable, thin and non-resilient, visible flabby, there are obvious pressing trace. |
| 8. | Skin Softness | Tactile | Very soft, easier to bend deformation by external forces but not saggy | Adequate to acceptable soft | Perceivable to slight soft | Very stiff, hard to bend deformation by external forces |
| 9. | Skin Smoothness | Tactile | No roughness of the treatment area; skin is completely smooth and pliable | Some areas of slightly irregular roughness in the treatment area | Several areas of definite roughness of the treatment area | Very rough, significantly (severe) palpable skin roughness, drag and/or surface bumps/depressions, mark roughness of the treatment area associated with a stiff feeling |
| 10. | PIH | Visual | Very light, similar to the bare skin | / | / | Very dark, very significant |
| 11. | Skin fairness | Visual | Very fair skin color | / | / | Very dark skin color |
| 12. | Overall hyperpigmentation | Visual | No hyperpigmentation, skin clarity | / | / | Significantly (severe) hyperpigmentation, numerous spots, uneven skin tone |
| 13. | Global fine line | Visual | No fine lines present; skin looks completely smooth. | Rare, presence of fine lines that area widely spaced apart in the treatment area. | Moderate number of fine lines near each other in the treatment area. | Numerous, many fine lines densely packed together in the treatment area. |

**Supplementary Appendix F. Attributes of the physical symptom scale (0 to 4 scale)**

| **Attributes** | **Score 0**  **None** | **Score I**  **Very Slight** | **Score 2**  **Slight** | **Score 3**  **Moderate** | **Score 4**  **Severe** |
| --- | --- | --- | --- | --- | --- |
| Dryness | No dryness | Superficial lines and/or white powdery appearance beginning to develop | Uniform dryness; scales beginning to separate and occasional uplifting scales | Uplifting scales; may be a few shallow cracks | Well-defined scaling, fissures and signs of  bleeding may be present; some erythema is present |
| Erythema | No erythema | Very faint erythema (skin has a very light pink color) | Faint erythema (skin reaction is more apparent with clear borders but is still pink with more intensity | Bright erythema  (erythema is apparent in bright pink and borders are clearly defined) | Very bright erythema (skin is bright red, borders arc very well defined capillaries and bruising may be  visible |
| Pimple | No pimple | Comedones only or less than 10 lcsions, nonc on trunk, no scars, non-inflammatory lesion | 10-25 papulcs, mild scarring, inflammatory lesion (<5 mm) | Pustules, moderate scarring | Nodules or cysts, extensive scarring,  inflammatory lesion (>5 mm) |
| Desquamation | No desquamation | Patchy, slight powderiness with small scales | General, slight  powderiness with  small lifting scales | General, moderate powderiness with cracking and scales | Severe, heavy powde1iness with cracking and lifting scales |
| Edema | No swelling | Very slight swelling, indistinct border | Defined swelling,  distinct border | Defined swelling, raise border (1 mm) | Pronounced swelling, raised border (> 1 mm) |

**Supplementary Appendix G. Diagram 1 skin measurement**

**Supplementary Appendix H. Self-reported questionnaire**

|  | Question | Timm | T4w | T8w | T12w |
| --- | --- | --- | --- | --- | --- |
| Skin quality | Skin feels softer |  |  |  |  |
|  | Skin feels smoother |  |  |  |  |
|  | Skin looks more radiant |  |  |  |  |
|  | Skin texture looks more refined |  |  |  |  |
|  | Pores looks less visible |  |  |  |  |
|  | Skin looks more translucent |  |  |  |  |
|  | Skin feels more plumpy |  |  |  |  |
|  | Skin feels more elastic |  |  |  |  |
|  | Skin looks with healthy glow |  |  |  |  |
|  | Skin tone looks more even |  |  |  |  |
|  | Skin looks less yellowish |  |  |  |  |
|  | Dark spots look less visible |  |  |  |  |
| Hydration/Oil  control | Skin feels hydrated |  |  |  |  |
|  | Skin feels oil water balance |  |  |  |  |
| Anti-aging | Skin feels firmer |  |  |  |  |
|  | Fine lines look less visible |  |  |  |  |
|  | Wrinkles look less visible* |  |  |  |  |
|  | Skin feels more vitalized (energetic) |  |  |  |  |
|  | Skin looks younger |  |  |  |  |
|  | Skin looks aging prevention |  |  |  |  |
| Cosmeticity | The product is pleasant to use | / |  | / | |
|  | The product doesn’t leave the skin sticky |  |  |  |  |
|  | The product is quickly absorbed |  |  |  |  |
|  | Boost fast absorption of following skincare routine |  |  |  |  |
|  | The product is gentle for the skin |  |  |  |  |
|  | The product is easy to spread |  |  |  |  |
|  | The texture of the product is not greasy |  |  |  |  |
|  | The texture of the product is not sticky |  |  |  |  |
|  | The product does not leave the skin shiny |  |  |  |  |
|  | The texture of the product is comfortable |  |  |  |  |
|  | This product is suitable for my skin type |  |  |  |  |

**Table S1. PIH of acne mark scale in the younger group**

|  |  | Soft peeling serum group  (N=31) | Benchmark serum group  (N=30) | P-value |
| --- | --- | --- | --- | --- |
| PIH of acne mark scale | T0 | 3.11±0.14 | 3.20±0.13 |  |
|  | T4w, P | 2.44±0.16, 0.013 | 2.57±0.11, 0.002 |  |
|  | Change from T0 to T4w | -0.68±0.09 | -0.63±0.08 | 0.717 |
|  | T8w, P | 2.19±0.17, <0.001 | 2.25±0.12, <0.001 |  |
|  | Change from T0 to T8w | -0.92±0.09 | -0.95±0.11 | 0.880 |
|  | T12w, P | 1.77±0.18, <0.001 | 1.80±0.12, <0.001 |  |
|  | Change from T0 to T12w | -1.34±0.13 | -1.40±0.14 | 0.947 |

**Table S2.** **Skin tone attributes**

|  |  | Younger group | | | Older group | | |
| --- | --- | --- | --- | --- | --- | --- | --- |
|  |  | Soft peeling serum (N=31) | Benchmark serum (N=30) | P | Soft peeling serum (N=32) | Benchmark serum (N=31) | P |
| Skin radiance | T0 | 4.94±0.09 | 4.88±0.07 |  | 4.80±0.06 | 4.84±0.07 |  |
|  | T4w, P | 4.45±0.07, <0.001 | 4.50±0.07, 0.004 |  | 4.42±0.07, 0.001 | 4.50±0.05, 0.001 |  |
|  | Change from T0 to T4w | -0.48±0.04 | -0.38±0.06 | 0.140 | -0.38±0.06 | -0.34±0.05 | 0.804 |
|  | T8w, P | 4.26±0.07, <0.001 | 4.28±0.08, <0.001 |  | 4.13±0.06, <0.001 | 4.24±0.05, <0.001 |  |
|  | Change from T0 to T8w | -0.68±0.07 | -0.60±0.08 | 0.472 | -0.67±0.07 | -0.60±0.06 | 0.536 |
|  | T12w, P | 3.95±0.08, <0.001 | 4.07±0.07, <0.001 |  | 3.84±007, <0.001 | 4.00±0.06, <0.001 |  |
|  | Change from T0 to T12w | -0.98±0.08 | -0.82±0.08 | 0.159 | -0.95±0.07 | -0.84±0.06 | 0.308 |
| Skin translucency | T0 | 4.76±0.11 | 4.78±0.11 |  | 5.16±0.09 | 5.06±0.09 |  |
|  | T4w, P | 4.24±0.11, 0.005 | 4.38±0.12, 0.044 |  | 4.70±0.10, 0.004 | 4.69±0.11, 0.067 |  |
|  | Change from T0 to T4w | -0.52±0.02 | -0.40±0.04 | 0.006 | -0.45±0.04 | -0.37±0.04 | 0.173 |
|  | T8w, P | 3.89±0.12, <0.001 | 4.03±0.12, <0.001 |  | 4.42±0.10, <0.001 | 4.42±0.12, <0.001 |  |
|  | Change from T0 to T8w | -0.87±0.05 | -0.75±0.05 | 0.077 | -0.73±0.05 | -0.65±0.07 | 0.453 |
|  | T12w, P | 3.52±0.12, <0.001 | 3.68±0.11, <0.001 |  | 4.02±0.10, <0.001 | 3.90±0.12, <0.001 |  |
|  | Change from T0 to T12w | -1.24±0.07 | -1.10±0.05 | 0.089 | -1.14±0.06 | -1.16±0.07 | 0.825 |
| Skin fairness | T0 | 4.74±0.12 | 4.68±0.12 |  | 5.08±0.10 | 5.00±0.10 |  |
|  | T4w, P | 4.26±0.12, 0.017 | 4.35±0.13, 0.214 |  | 4.72±0.11, 0.043 | 4.71±0.12, 0.222 |  |
|  | Change from T0 to T4w | -0.48±0.03 | -0.33±0.04 | 0.006 | -0.36±0.05 | -0.29±0.05 | 0.313 |
|  | T8w, P | 3.97±0.12, <0.001 | 4.12±0.14, 0.009 |  | 4.50±0.10, <0.001 | 4.47±0.11, 0.005 |  |
|  | Change from T0 to T8w | -0.77±0.06 | -0.57±0.07 | 0.054 | -0.58±0.05 | -0.53±0.06 | 0.563 |
|  | T12w, P | 3.77±0.13, <0.001 | 3.83±0.13, <0.001 |  | 4.14±0.10, <0.001 | 4.10±0.12, <0.001 |  |
|  | Change from T0 to T12w | -0.97±0.07 | -0.85±0.09 | 0 393 | -0.94±0.07 | -0.90±0.07 | 0.600 |
| Skin tone evenness | T0 | 4.85±0.10 | 4.82±0.10 |  | 5.19±0.10 | 5.19±0.09 |  |
|  | T4w, P | 4.48±0.10, 0.044 | 4.52±0.11, 0.130 |  | 4.86±0.11, 0.111 | 4.89±0.12, 0.178 |  |
|  | Change from T0 to T4w | -0.37±0.05 | -0.30±0.05 | 0.300 | -0.33±0.04 | -0.31±0.04 | 0.723 |
|  | T8w, P | 4.21±0.10, <0.001 | 4.32±0.10, 0.003 |  | 4.64±0.11, 0.002 | 4 60±0.12, 0.001 |  |
|  | Change from T0 to T8w | -0.65±0.07 | -0.50±0.05 | 0.096 | -0.55±0.05 | -0.60±0.06 | 0.486 |
|  | T12w, P | 3.92±0.11, <0.001 | 4 05±0.10, <0.001 |  | 4.36±0.11, <0.001 | 4.34±0.13, <0.001 |  |
|  | Change from T0 to T12w | -0.94±0.08 | -0.77±0.07 | 0.174 | -0.83±0.07 | -0.85±0.07 | 0.870 |
| Overall hyperpigmentation | T0 | 4.45±0.11 | 4.48±0.13 |  | 5.00±0.11 | 4.94±0.10 |  |
|  | T4w, P | 4.06±0.12, 0.085 | 4.22±0.14, 0.450 |  | 4.69±0.13, 0.264 | 4.65±0.12, 0.336 |  |
|  | Change from T0 to T4w | -0.39±0.05 | -0.27±0.05 | 0.094 | -0.31±0.06 | -0.29±0.05 | 0.951 |
|  | T8w, P | 3.71±0.13, <0.001 | 3.92±0.14, 0.015 |  | 4.41±0.14, 0.006 | 4.31±0.15, 0.003 |  |
|  | Change from T0 to T8w | -0.74±0.07 | -0.57±0.08 | 0.134 | -0.59±0.09 | -0.63±0.08 | 0.593 |
|  | T12w, P | 3.27±0.13, <0.001 | 3.57±0.14, <0.001 |  | 4.11±0.15, <0.001 | 3.98±0.16, <0.001 |  |
|  | Change from T0 to T12w | -1.18±0.08 | -0.92±0.08 | 0.030 | -0.89±0.09 | -0.95±0.09 | 0.568 |
| Skin dullness | T0 | 4.68±0.14 | 4.55±0.14 |  | 5.08±0.10 | 4.98±0.10 |  |
|  | T4w, P | 4.15±0.14, 0.018 | 4.18±0.14, 0.207 |  | 4.66±0.10, 0.013 | 4.60±0.10, 0.050 |  |
|  | Change from T0 to T4w | -0.53±0.02 | -0.37±0.04 | 0.001 | -0.42±0.04 | -0.39±0.04 | 0.555 |
|  | T8w, P | 3.76±0.12, <0.001 | 3.90±0.14, 0.005 |  | 4.34±0.10, <0.001 | 4.31±0.11, <0.001 |  |
|  | Change from T0 to T8w | -0.92±0.05 | -0.65±0.07 | 0.007 | -0.73±0.06 | -0.68±0.06 | 0.391 |
|  | T12w, P | 3.40±0.13, <0.001 | 3.58±0.14, <0.001 |  | 3.95±0.11, <0.001 | 3.87±0.13, <0.001 |  |
|  | Change from T0 to T12w | -1.27±0.06 | -0.97±0.08 | 0.007 | -1.13±0.07 | -1.11±0.07 | 0.750 |

**Table S3.** **Skin quality attributes**

|  |  | Younger group | | | Older group | | |
| --- | --- | --- | --- | --- | --- | --- | --- |
|  |  | Soft peeling serum (N=31) | Benchmark serum (N=30) | P | Soft peeling serum (N=32) | Benchmark serum (N=31) | P |
| Skin smoothness (tactile) | T0 | 4.40±0.12 | 4.42±0.10 |  | 4.36±0.08 | 4.35±0.08 |  |
|  | T4w, P | 3.87±0.12, 0.007 | 4.03±0.09, 0.047 |  | 3.73±0.09, <0.001 | 3.92±0.08, 0.001 |  |
|  | Change from T0 to T4w | -0.53±0.05 | -0.38±0.06 | 0.042 | -0.63±0.06 | -0.44±0.04 | 0.020 |
|  | T8w, P | 3.69±0.12, <0.001 | 3.87±0.11, 0.002 |  | 3.38±0.08, <0.001 | 3.55±0.08, <0.001 |  |
|  | Change from T0 to T8w | -0.71±0.08 | -0.55±0.09 | 0.221 | -0.98±0.07 | -0.81±0.08 | 0.105 |
|  | T12w, P | 3.39±0.12, <0.001 | 3.40±0.14, <0.001 |  | 3.14±0.08, <0.001 | 3.24±0.10, <0.001 |  |
|  | Change from T0 to T12w | -1.02±0.10 | -1.02±0.12 | 0.997 | -1.22±0.07 | -1.11±0.09 | 0.389 |
| Skin elasticity | T0 | 3.34±0.05 | 3.45±0.06 |  | 3.94±0.08 | 3.87±0.06 |  |
|  | T4w, P | 3 03±0.06, 0.002 | 3.12±0.07, 0.002 |  | 3.55±0.09, 0.002 | 3.60±0.07, 0.027 |  |
|  | Change from T0 to T4w | -0.31±0.04 | -0.33±0.04 | 0.665 | -0.39±0.04 | -0.27±0.05 | 0.052 |
|  | T8w, P | 2.82±0.06, <0.001 | 2.90±0.07, <0.001 |  | 3.38±0.08, <0.001 | 3.44±0.09, <0.001 |  |
|  | Change from T0 to T8w | -0.52±0.06 | -0.55±0.06 | 0.685 | -0.56±0.06 | -0.44±0.06 | 0.134 |
|  | T12w, P | 2.77±0.06, <0.001 | 2 80±0.07, <0.001 |  | 3.25±0.07, <0.001 | 3.37±0.07, <0.001 |  |
|  | Change from T0 to T12w | -0.56±0.06 | -0.65±0.06 | 0.432 | -0.69±0.04 | -0.50±0.07 | 0.015 |
| Skin softness | T0 | 3.98±0.09 | 3.95±0.08 |  | 3.95±0.08 | 3.89±0.07 |  |
|  | T4w, P | 3.52±0.09, 0.002 | 3.60±0.09, 0.013 |  | 3.48±0.09, <0.001 | 3.52±0.08, 0.003 |  |
|  | Change from T0 to T4w | -0.47±0.06 | -0.35±0.05 | 0.146 | -0.47±0.05 | -0.37±0.04 | 0.159 |
|  | T8w, P | 3.31±0.09, <0.001 | 3.43±0.09, <0.001 |  | 3.17±0.09, <0.001 | 3.15±0.07, <0.001 |  |
|  | Change from T0 to T8w | -0.68±0.09 | -0.52±0.07 | 0.077 | -0.78±0.07 | -0.74±0.08 | 0.514 |
|  | T12w, P | 3.02±0.10, <0.001 | 3.13±0.08, <0.001 |  | 2.97±0.09, <0.001 | 2.81±0.08, <0.001 |  |
|  | Change from T0 to T12w | -0.97±0.11 | -0.82±0.07 | 0.154 | -0.98±0.08 | -1.08±0.07 | 0.518 |
| Skin firmness | T0 | 3.89±0.07 | 4.07±0.10 |  | 4.72±0.08 | 4.76±0.07 |  |
|  | T4w, P | 3.44±0.07, <0.001 | 3.78±0.09, 0.097 |  | 4.28±0.09, 0.001 | 4.40±0.07, 0.006 |  |
|  | Change from T0 to T4w | -0.45±0.04 | -0.28±0.05 | 0.006 | -0.44±0.04 | -0.35±0.04 | 0.194 |
|  | T8w, P | 3.13±0.08, <0.001 | 3.45±0.08, <0.001 |  | 3.91±0.08, <0.001 | 4.00±0.08, <0.001 |  |
|  | Change from T0 to T8w | -0.76±0.07 | -0.62±0.07 | 0.132 | -0.81±0.05 | -0.76±0.06 | 0.653 |
|  | T12w, P | 2.89±0.08, <0.001 | 3.15±0.09, <0.001 |  | 3 70±0.08, <0.001 | 3.77±0.09, <0.001 |  |
|  | Change from T0 to T12w | -1.00±0.07 | -0.92±0.08 | 0.513 | -1.02±0.06 | -0.98±0.07 | 0.817 |
| Skin plumpness | T0 | 3 81±0.08 | 3.98±0.08 |  | 4.67±0.09 | 4.73±0.08 |  |
|  | T4w, P | 3.47±0.08, 0.011 | 3.63±0.09, 0.020 |  | 4.23±0.10, 0.004 | 4.37±0.08, 0.028 |  |
|  | Change from T0 to T4w | -0.34±0.04 | -0.35±0.04 | 0.850 | -0.44±0.04 | -0.35±0.04 | 0.150 |
|  | T8w, P | 3.19±0.09, <0.001 | 3.30±0.09, <0.001 |  | 3.89±0.09, <0.001 | 4.02±0.10, <0.001 |  |
|  | Change from T0 to T8w | -0.61±0.06 | -0.68±0.06 | 0.390 | -0.78±0.05 | -0.71±0.06 | 0.643 |
|  | T12w, P | 2.94±0.08, <0.001 | 3.05±0.09, <0.001 |  | 3 66±0.09, <0.001 | 3.77±0.10, <0.001 |  |
|  | Change from T0 to T12w | -0.87±0.07 | -0.93±0.07 | 0.431 | -1.02±0.07 | -0.95±0.06 | 0.473 |
| Cheek pores | T0 | 4.33±0.13 | 4.20±0.13 |  | 4.46±0.13 | 4.42±0.12 |  |
|  | T4w, P | 3.99±0.14, 0.185 | 3.89±0.12, 0.194 |  | 4.05±0.13, 0.081 | 4.12±0.12, 0.247 |  |
|  | Change from T0 to T4w | -0.34±0.03 | -0.33±0.03 | 0.630 | -0.40±0.03 | -0.30±0.03 | 0.012 |
|  | T8w, P | 3.70±0.13, 0.002 | 3.67±0.11, 0.008 |  | 3.79±0.12, 0.001 | 3.88±0.12, 0.008 |  |
|  | Change from T0 to T8w | -0.63±0.04 | -0.53±0.05 | 0.113 | -0.67±0.05 | -0.54±0.04 | 0.049 |
|  | T12w, P | 3.47±0.12, <0.001 | 3.39±0.11, <0.001 |  | 3.44±0.12, <0.001 | 3.61±0.11, <0.001 |  |
|  | Change from T0 to T12w | -0.86±0.05 | -0.81±0.05 | 0.492 | -l.02±0.07 | -0.81±0.05 | 0.013 |

**Table S4. Wrinkle attributes**

|  |  | Younger group | | | Older group | | |
| --- | --- | --- | --- | --- | --- | --- | --- |
|  |  | Soft peeling serum (N=31) | Benchmark serum (N=30) | P | Soft peeling serum (N=32) | Benchmark serum (N=31) | P |
| Crow’s feet wrinkles | T0 | 0.91±0.07 | 1.00±0.10 |  | 2.61±0.12 | 2.75±0.12 |  |
|  | T4w, P | 0.74±0.06, 0.150 | 0.87±0.09, 0.710 |  | 2.32±0.12, 0.273 | 2.51±0.12, 0.475 |  |
|  | Change from T0 to T4w | -0.16±0.03 | -0.13±0.03 | 0.351 | -0.29±0.04 | -0.25±0.03 | 0.370 |
|  | T8w, P | 0.60±0.05, 0.001 | 0.78±0.09, 0.267 |  | 2.10±0.13, 0.015 | 2.32±0.14, 0.063 |  |
|  | Change from T0 to T8w | -0.30±0.04 | -0.22±0.04 | 0.141 | -0.51±0.05 | -0.44±0.05 | 0.315 |
|  | T12w, P | 0.53±0.05, <0.001 | 0.66±0.08, 0.034 |  | 1.86±0.12, <0.001 | 2.18±0.13, 0.008 |  |
|  | Change from T0 to T12w | -0.38±0.05 | -0.34±0.05 | 0.580 | -0.75±0.06 | -0.57±0.06 | 0.036 |
| Forehead wrinkles | T0 | 0.79±0.07 | 0.97±0.09 |  | 2.16±0.05 | 2.26±0.11 |  |
|  | T4w, P | 0.59±0.05, 0.013 | 0.80±0.08, 0.279 |  | 1.78±0.06, <0.001 | 1.94±0.11, 0.140 |  |
|  | Change from T0 to T4w | -0.21±0.04 | -0.17±0.04 | 0.489 | -0.38±0.04 | -0.32±0.03 | 0.451 |
|  | T8w, P | 0.49±0.03, <0.001 | 0.67±0.07, 0.013 |  | 1.47±0.07, <0.001 | 1.59±0.10, <0.001 |  |
|  | Change from T0 to T8w | -0.30±0.05 | -0.31±0.06 | 0.887 | -0.69±0.05 | -0.68±0.05 | 0.785 |
|  | T12w, P | 0.44±0.03, <0.001 | 0.55±0.06, <0.001 |  | 1.31±0.08, <0.001 | 1.35±0.11, <0.001 |  |
|  | Change from T0 to T12w | -0.35±0.06 | -0.42±0.06 | 0.509 | -0.85±0.06 | -0.91±0.06 | 0.511 |
| Small folds on nasolabial zone | T0 | 1.18±0.05 | 1.39±0.10 |  | 2.81±0.08 | 2.88±0.08 |  |
|  | T4w, P | 0.88±0.05, <0.001 | 1.06±0.10, 0.046 |  | 2.35±0.07, <0.001 | 2.50±0.08, 0.010 |  |
|  | Change from T0 to T4w | -0.30±0.03 | -0.33±0.03 | 0.459 | -0.45±0.03 | -0.38±0.04 | 0.094 |
|  | T8w, P | 0.69±0.04, <0.001 | 0.94±0.09, 0.003 |  | 2.05±0.08, <0.001 | 2.22±0.09, <0.001 |  |
|  | Change from T0 to T8w | -0.49±0.04 | -0.46±0.06 | 0.608 | -0.76±0.04 | -0.66±0.05 | 0.120 |
|  | T12w, P | 0.58±0.04, <0.001 | 0.81±0.09, <0.001 |  | 1.80±0.08, <0.001 | 2.00±010, <0.001 |  |
|  | Change from T0 to T12w | -0.60±0.03 | -0.59±0.07 | 0.600 | -1.01±0.06 | -0.88±0.07 | 0.131 |
| Global fine lines | T0 | 2.52±0.09 | 2.65±0.10 |  | 3.59±0.12 | 3.55±0.10 |  |
|  | T4w, P | 1.82±0.08, <0.001 | 2.12±0.10, <0.001 |  | 2.92±0.12, <0.001 | 3.00±0.10, 0.002 |  |
|  | Change from T0 to T4w | -0.69±0.05 | -0.53±0.05 | 0.002 | -0.67±0.06 | -0.55±0.04 | 0.094 |
|  | T8w, P | 1.34±0.07, <0.001 | 1.65±0.09, <0.001 |  | 2.41±0.10, <0.001 | 2.39±0.09, <0.001 |  |
|  | Change from T0 to T8w | -1.18±0.08 | -1.00±0.08 | 0.079 | -1.19±0.07 | -l.16±0.07 | 0.631 |
|  | T12w, P | 1.07±0.08, <0.001 | 1.17±0.07, <0.001 |  | 1.86±0.08, <0.001 | 1.97±0.09, <0.001 |  |
|  | Change from T0 to T12w | -1.45±0.10 | -1.48±0.07 | 0.643 | -1.73±0.09 | -1.58±0.09 | 0.154 |

**Figure S1. The fluorescence evaluation images in dansyl chloride patch test**

**Figure S2. Cumulative fluorescence scores in clinical study 1**

**Figure S3. Skin hydration, TEWL and elastic properties trend**

**Figure S4. Trends in skin attributes over time in the older group**

**Figure S5. Skin hydration, TEWL and elastic properties trend**
